# *QuickStats:* Percentage of Residential Care Communities* That Use Electronic Health Records, by Community Bed Size — United States, 2018, 2020, and 2022^†^

**DOI:** 10.15585/mmwr.mm7317a6

**Published:** 2024-05-02

**Figure:**
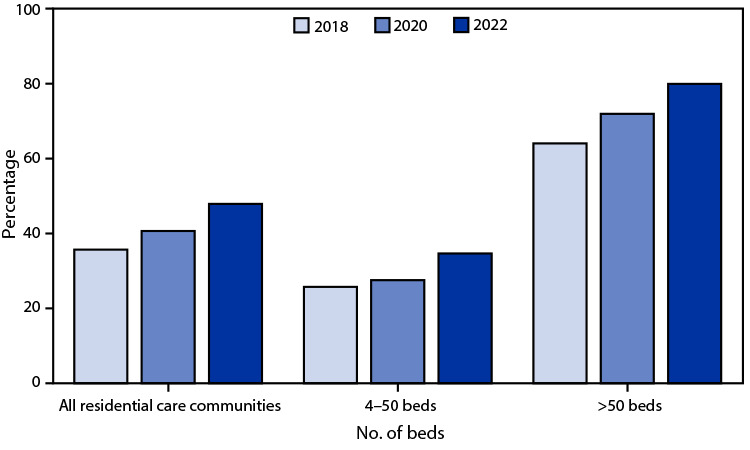
From 2018 to 2022, the percentage of residential care communities (RCCs) using electronic health records (EHRs) increased from 36% to 48%. Use of EHRs increased during this time regardless of RCC size, and larger RCCs were more likely to use EHRs compared with smaller RCCs.

**Supplementary Table:** https://stacks.cdc.gov/view/cdc/153378

**Source:** National Center for Health Statistics, National Post-acute and Long-term Care Study, 2018, 2020, and 2022 data. https://www.cdc.gov/nchs/npals/questionnaires.htm

